# Retrograde Lymphatic Spread of Esophageal Cancer: A Case Report

**DOI:** 10.1097/MD.0000000000001139

**Published:** 2015-07-13

**Authors:** Hisashi Oshiro, Yoshiaki Osaka, Shingo Tachibana, Takaya Aoki, Takayoshi Tsuchiya, Toshitaka Nagao

**Affiliations:** From the Departments of Anatomic Pathology (HO, TN), Gastrointestinal and Pediatric Surgery (YO, ST), and Gastroenterology and Hepatology (TA, TT), Tokyo Medical University, Tokyo, Japan.

## Abstract

The concept of the retrograde lymphatic spread of cancer cells appears to account for a subset of the essential mechanisms of cancer metastasis in various organs. However, no adequate data currently exist to illustrate the pathology of the retrograde lymphatic metastasis of cancer cells in human bodies. To shed light on this phenomenon, we report a case of a 63-year-old Japanese man who underwent an esophagectomy and lymph node dissection for early-stage esophageal cancer.

The patient's clinical information was evaluated by board-certified surgeons and internists. Surgically excised materials were histopathologically evaluated by attending pathologists.

Postoperative pathological examination revealed that the patient's tumor was a well-differentiated squamous cell carcinoma with negative surgical margins (T1N0M0, stage I). Apart from the primary lesion, a single lymphatic vessel invasion was found between the lamina propria and lamina muscularis of the esophagus where intralymphatic cancer cells had spread against the direction of backflow prevention valves and skipped beyond these valves without destroying them.

The present case demonstrated that the retrograde lymphatic spread of cancer cells can occur in valve-equipped lymphatic vessels. Our study may not only provide a scientific basis for the concept of retrograde lymphatic metastasis but also explain a portion of the complexities associated with the lymphogenous metastasis of esophageal cancer.

## INTRODUCTION

Lymph node (LN) metastasis is an important prognostic predictor for esophageal squamous cell carcinoma.^1^ The bidirectional or skip nodal spread of cancer cells is thought to be a key feature of lymphatic metastasis in patients with thoracic esophageal squamous cell carcinoma.^1,2^ Thus, it is important to discover the mechanisms of the lymphatic metastasis of esophageal cancer.

The concept of the retrograde lymphatic spread of cancer cells appears to account for a subset of the essential mechanisms of cancer metastasis in not only the esophagus but also various other regions, such as the lungs, pleura, mediastinum, heart, pericardium, axilla, spleen, retroperitoneum, perinephritic space, testis, ovary, prostate, penis, and vulva.^3–5^ However, questions remain regarding the term *retrograde lymphatic metastasis*, which appears in the literature but may sometimes be incorrectly used to describe anterograde lymphatic metastasis to an unusual site due to anatomical variation in the lymphatic distribution and/or caused by the alteration of the lymphatic distribution by a particular pathological condition.

An anterograde lymph flow can be defined as a flow in the direction of a lymphatic valve, and a retrograde lymph flow can be defined as a flow against the direction of a lymphatic valve. To the best of our knowledge, if these definitions are rigorously followed, no adequate data currently exist to illustrate the pathology of the retrograde lymphatic metastasis of cancer cells in human bodies. Thus, our central question is whether the retrograde lymphatic spread of cancer cells actually occurs in human bodies.

In this report, we describe an illustrative case of esophageal cancer in which cancer cells exhibited retrograde lymphatic spread. We also discuss the mechanisms and significance of this phenomenon.

## METHODS

Written informed consent for this case study was obtained from the patient. The patient's clinical information was evaluated by board-certified surgeons (YO and ST) and internists (TA and TT). Surgically excised materials were immediately fixed in 20% neutral buffered formalin for routine histological examination and evaluated by attending pathologists (HO and TN). Immunohistochemistry was performed using a D2-40 antibody (D2-40; Nichirei Bioscience; Tokyo, Japan), a detection kit (Histofine Simple Stain MAX PO, MULTI; Nichirei Bioscience), and an autostainer (Histostainer; Nichirei Bioscience) according to the manufacturer's instruction.

## RESULTS

### Clinical Findings

**FIGURE 1:**
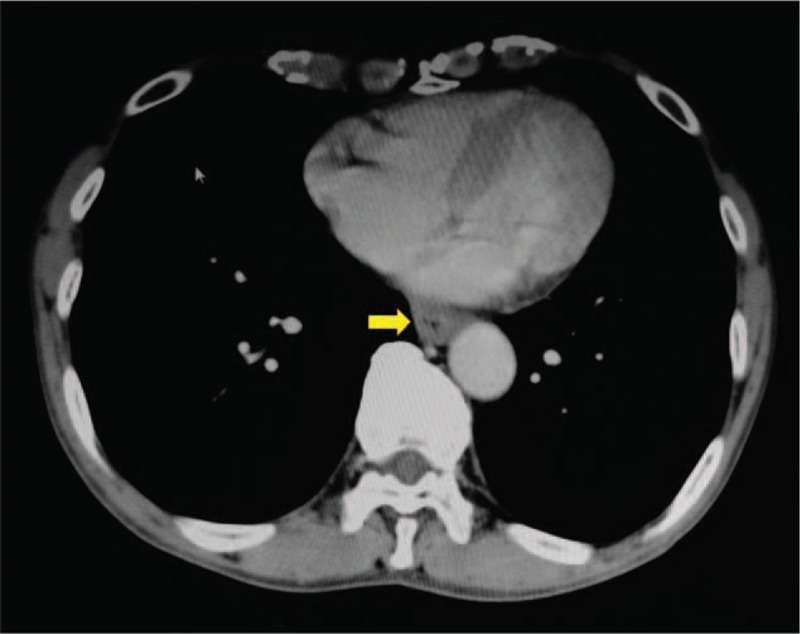
A contrast enhanced computed tomographic image of the thoracic organs demonstrates no remarkable changes, except for slight thickening of the wall in the lower thoracic portion of the esophagus (arrow).

A 63-year-old Japanese man was referred to our hospital for a detailed examination and treatment of esophageal cancer that had been detected during his health checkup. His history was unremarkable except for hypertension, which had been well controlled by an antihypertensive drug. He had been a smoker (Brinkman index = 1290) and had consumed approximately 34–54 g of alcohol per day for 43 years. His mother had suffered from gastric cancer, and his elderly brother had suffered from bladder cancer. No metastatic lesions were detected in his preoperative work-up, which consisted of systemic computed tomography (Figure 1) and ^18^F-fluorodeoxyglucose positron emission tomography.

The patient underwent subtotal esophagectomy and LN dissection with assistance from a da Vinci surgical system followed by a thoracoscopy and anastomosis of the stomach to the cervical esophagus via the posterior portion of the mediastinum, with the gastric tube reconstructed by hand-assisted laparoscopic surgery. His postoperative course was uneventful. He has been regularly followed without adjuvant therapy and has remained free from disease recurrence for 38 months since his operation.

### Pathological Findings

**FIGURE 2:**
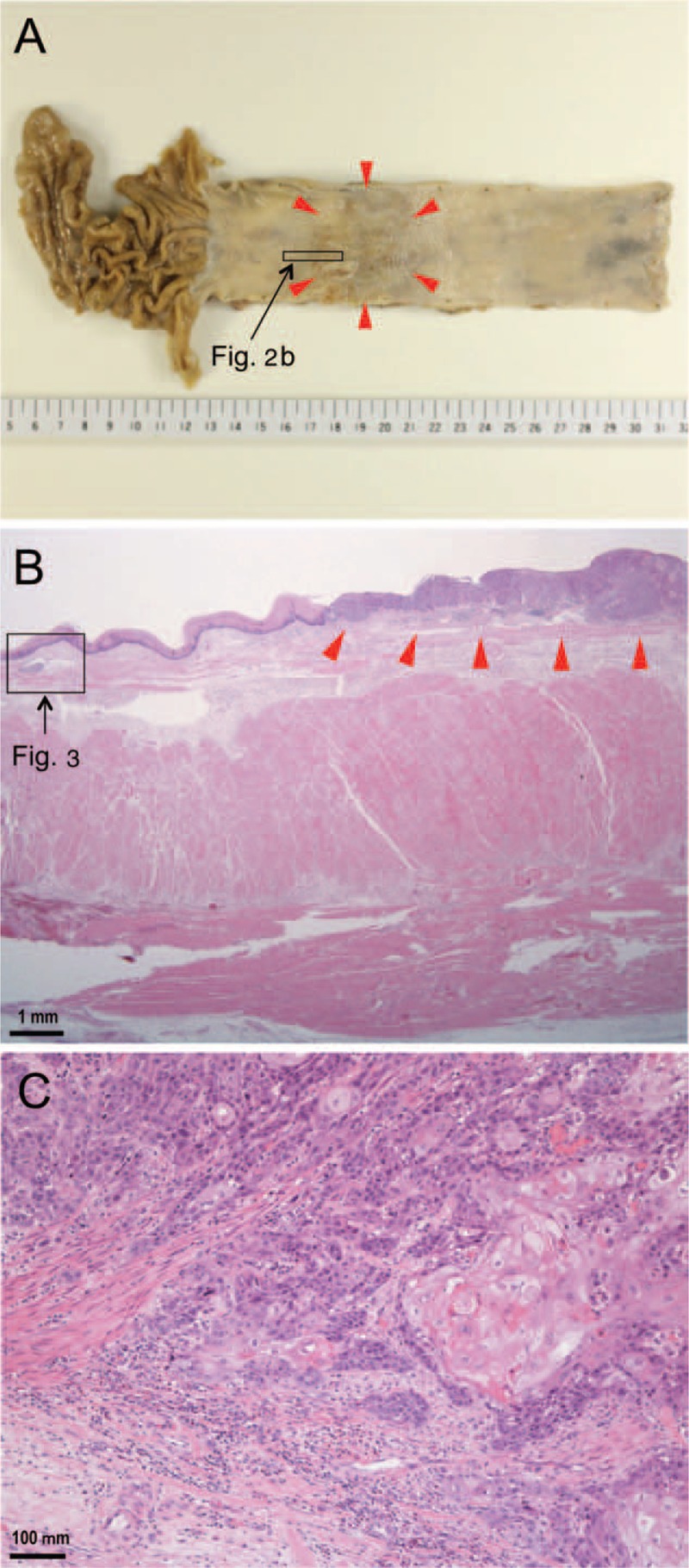
Pathological findings for the examined esophageal cancer. A: A gross image depicts a superficial (0 – Ip + IIc type) single tumor 47 × 38 mm in size located in the middle to the lower thoracic portion of the esophagus (arrowheads). B: A low-power photomicrograph reveals a primary lesion (arrowheads) and an area of retrograde lymphatic invasion distant from the primary lesion (rectangle) (hematoxylin and eosin stain). C: A high-power photomicrograph illustrates the primary lesion and features indicating the invasion of well-differentiated squamous cell carcinoma into the submucosal layer (hematoxylin and eosin stain).

**FIGURE 3:**
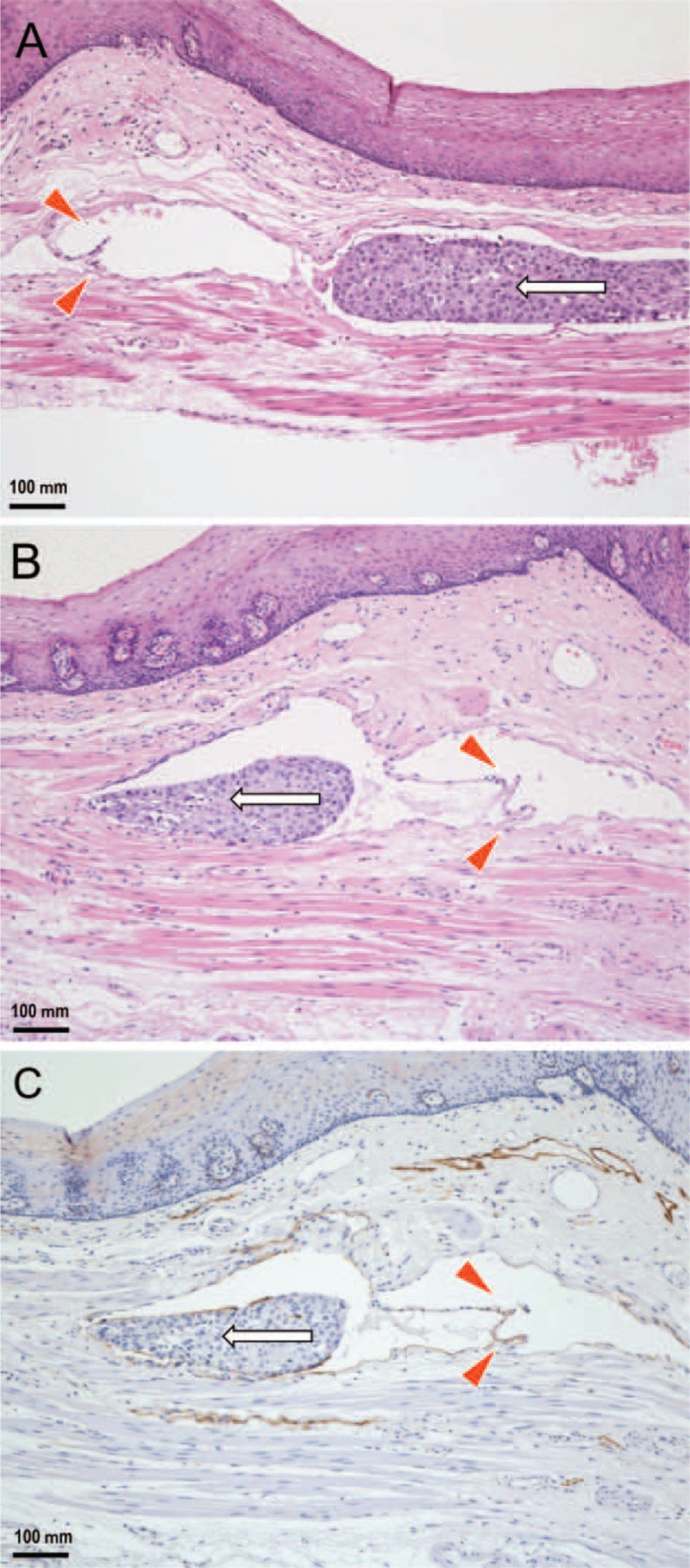
Histopathological findings regarding the retrograde lymphatic spread of esophageal cancer cells (enlargement of the rectangular area in Figure 2B). A: Distant from the primary lesion, a single lymphatic vessel running along the lamina muscularis has been invaded by cancer cells. Intralymphatic cancer cells (arrow) are spread against the direction of backflow prevention valves (arrowheads). B: A serial tissue section reveals that intralymphatic cancer cells (arrow) skipped beyond the valves (arrowheads) without morphological destruction of the valves (hematoxylin and eosin stain). C: The endothelium is positive for D2-40, demonstrating that the depicted tissue sample is indeed a lymphatic vessel that has been invaded by cancer cells (arrow) (immunohistochemistry).

After the operation, histopathological examination was performed on the excised esophagus and dissected LNs by total segmentation, and a final diagnosis of the lesion was generated (Figure 2). This lesion was a single tumor 47 × 38 mm in size located in the middle to the lower thoracic portion of the esophagus. The tumor was a superficial (0 – Ip + IIc type), well-differentiated squamous cell carcinoma with invasion into 2/3 of the depth of the submucosal layer (500 μm) from the inferior border of the muscularis mucosae, and it had negative surgical margins, no intramural metastasis and no LN metastases (pN0; #1: right cardiac LNs [0/3]; #3: LNs along the lesser curvature [0/9]; #7: LNs along the left gastric artery [0/3]; #101L: left cervical paraesophageal LN [0/5]; #105: upper thoracic paraesophageal LNs [0/5]; #106recL: left recurrent nerve LNs [0/3]; #106tbL: left tracheobronchial LNs [0/0]; #107: subcarinal LNs [0/4]; #108: middle thoracic paraesophageal LNs [0/2]; #109L: left main bronchus LNs [0/2]; #109R: right main bronchus LNs [0/8]; #110: lower thoracic paraesophageal LNs [0/1]; and #112: posterior mediastinal LNs [0/0]). The tumor was categorized as T1bN0M0 and stage I using the Japanese classification of esophageal cancer.^6^ Venous invasion was confirmed in 1 small vein in the esophagus by Elastica-van Gieson staining. In particular, distant from the primary lesion, a single lymphatic vessel running along the lamina muscularis had been invaded by cancer cells (Figure 3). The nature of lymphatics was immunohistochemically confirmed using a D2-40 antibody. In this lymphatic vessel, 2 cancer cell clusters were observed. Notably, intralymphatic cancer cells had spread against the direction of backflow prevention valves. Moreover, serial tissue sections revealed that intralymphatic cancer cells had skipped beyond these valves without morphological destruction of the valves. The lymphatic lumen was dilated, and the cancer cell clusters appeared to be partially attached to the endothelium. The distance from the primary lesion to the distal margin of the intralymphatic cancer cell cluster was approximately 5 mm. The upward lymphatic vessel located after the cancer cell cluster that could be tracked by the tissue sections was approximately 100 μm in length, and it became smaller in diameter, accompanied by a closed lymphatic valve, and was no longer invaded by cancer cells.

## DISCUSSION

In the present case, one could consider the possibility of the anterograde lymphatic spread of cancer cells from other lymphatics. However, this possibility was excluded for the following reasons: the esophagus was histopathologically inspected by total segmentation, and only a single lymphatic vessel had been invaded by cancer cells; there was no LN metastasis; and no neoadjuvant therapy was administered to the patient. Moreover, the possibility of the artificial implantation of cancer cells was excluded for the following reasons: during the operation, surgeons gently excised the esophagus without strong pressure and after formalin fixation, pathologists gently cut the esophagus with a disposable blade without strong pressure, and the blade surface was cleaned with clear gauze following each cut.

**FIGURE 4:**
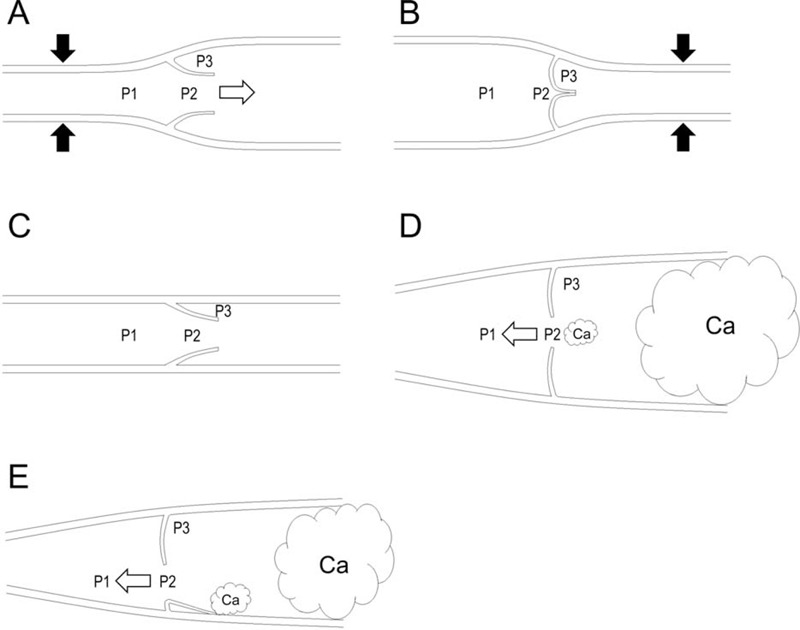
Schematic illustrations of lymph flows and fluid pressures at various points in a lymphatic vessel. The labeled pressures are in front of the valve (P1), in the funnel-shaped space between valve faces (P2), and in the pocket (P3). White arrows indicate the flow direction. Black arrows indicate an intrinsic or extrinsic lymphatic pump force. Ca indicates cancer cells. A: If P1 > P2 > P3, the valve is open, and fluid flows in an anterograde direction. B: If P1 = P2 < P3, the valve is closed. C: If P1 = P2 = P3, the stoppage of fluid flow occurs. D: If P1 < P2 = P3, the valve is open, and fluid flows in a retrograde direction. This situation can arise if P3 increases due to the lymphatic invasion of cancer cells and the regurgitation of lymphatic valves occurs due to lymphatic ectasia even if this regurgitation is not continuous. E: If P1 < P2 = P3, the valve is open, and fluid flows in a retrograde direction. This situation can arise if P3 increases due to the lymphatic invasion of cancer cells and the regurgitation of lymphatic valves occurs due to the adhesion of cancer cells to the endothelium even if this regurgitation is not continuous.

The mechanisms of lymphatic valve opening and closure can be depicted by considering the following fluid pressures at different locations around a valve^7^: the pressure in front of the valve (P1); the pressure in the funnel-shaped space between valve faces (P2); and the pressure in the pocket (P3) (Figure 4). If P1 > P2 > P3, the valve is open, and fluid flows in an anterograde direction (there are several other possibilities as well, including P1 > P2 = P3). If P1 = P2 < P3, the valve is closed. If P1 = P2 = P3, the stoppage of fluid flow occurs. Moreover, when P1 = P2 = P3, it is possible for particles, such as cancer cells, in the fluid to move through the valve by random Brownian diffusion. If P1 < P2 = P3, the valve is open, and fluid flows in a retrograde direction. Thus, the retrograde lymphatic spread of cancer cells can occur if P3 increases such that regurgitation of the valve occurs even if this regurgitation is not continuous.

The mechanisms of the retrograde lymphatic spread of cancer cells in the current case can be explained by the following phenomena: an increase in downstream intralymphatic pressure caused by lymphatic obstruction following cancer invasion or by an extrinsic lymph pump that depends on adjacent tissue movements, such as swallowing, esophageal peristalsis, arteriolar vasomotion, arteriolar pulsation, or respiration^7^; contractile dysfunction of the lymphatic wall caused by a tumor thrombus or by physiologically active substances, such as VEGF-C, which may have been released by cancer cells or lymphatic endothelial cells^8,9^; and intermittent regurgitation of the lymphatic valve caused by lymphatic ectasia or by the adhesion of cancer cells to lymphatic endothelial cells, presumably via adhesion-related molecules, such as ICAM-1, which have been expressed by either type of cell.^10,11^

The anatomical distribution in the middle and posterior mediastinal lymphatics is complex.^12^ Characteristically, lymphatics originating from the lower thoracic esophagus share with lymphatics originating from the pulmonary ligament, which has lymphatic stomata and a rich lymphatic network.^12–14^ These lymphatics connect with LNs of the tracheal bifurcation and with celiac LNs to form lymphatic chains.^15^ In addition, lymphatics originating from the thoracic esophagus frequently drain into the thoracic duct directly without intervening LNs.^12,13,16,17^ Notably, the human thoracic duct has multiple collateral branches (8.75 ± 3.89; mean ± SD) where anterograde 1-leaflet valves, retrograde 1-leaflet valves or oppositely arrayed 2-leaflet valves are frequently present, allowing for bidirectional lymph flow and accommodating the flow rate of the thoracic duct based on physiological or pathological conditions.^18,19^ These characteristics can account for part of the basis of bidirectional or skip nodal metastasis in esophageal cancer.

Recent experimental studies have demonstrated that the rerouting of lymph flow is a key feature in the development of lymphatic metastases.^8^ Larger metastases within the sentinel LN can obstruct lymphatic drainage, rerouting lymph flow toward another LN that is metastasis-free, which can lead to a false-negative sentinel LN.^20,21^ At a later stage, this LN accepts cancer cells via rerouted flow from the metastatic sentinel LN.^8^ However, the means by which lymphatic rerouting occurs remain incompletely elucidated. Certain investigators have speculated that the rerouting of lymph flow involves anterograde travel via newly generated bypassing and collateral lymphatics.^8^ In contrast, the present case study suggests that such rerouting can also occur via pre-existing lymphatic vessels in a retrograde manner.

There has been keen interest concerning the effect of extended lymphadenectomy and the usefulness of sentinel node navigation surgery for esophageal cancers among research-oriented clinical investigators.^22–26^ However, different procedures under different conditions by various institutions often make a comparison of studies difficult, leading to indefinite conclusions.^27^ We have more to accomplish before standardization of clinical practices against the lymphatic system. Further elucidation of human lymphatic pathology will help to develop and standardize better therapeutic strategies in esophageal cancers.

In summary, our study demonstrated that the retrograde lymphatic spread of cancer cells occurs against the direction of lymphatic valves without structural destruction of these valves. The present study may not only provide a scientific basis for the concept of retrograde lymphatic metastasis but also explain a portion of the complexities associated with the lymphogenous metastasis of esophageal cancer.
